# Supplementary material for: Brevity is not a universal in animal communication: evidence for compression depends on the unit of analysis in small ape vocalizations
Source: R Soc Open Sci. 2020 Apr 15;7(4):200151. doi: 10.1098/rsos.200151 (PMC7211885; doi:10.1098/rsos.200151)

Electronic supplementary material 1. The figures included in this ESM are representative unsupervised phrase classifications of male solos as determined using affinity propagation clustering. Each page has four 30-sec excerpts from male solos with boxes drawn around unique phrases, and the number above the box indicates phrase type. In cases where there are two pages with the same male identifier, this is the result of the solo spanning two separate 40-min recording windows.

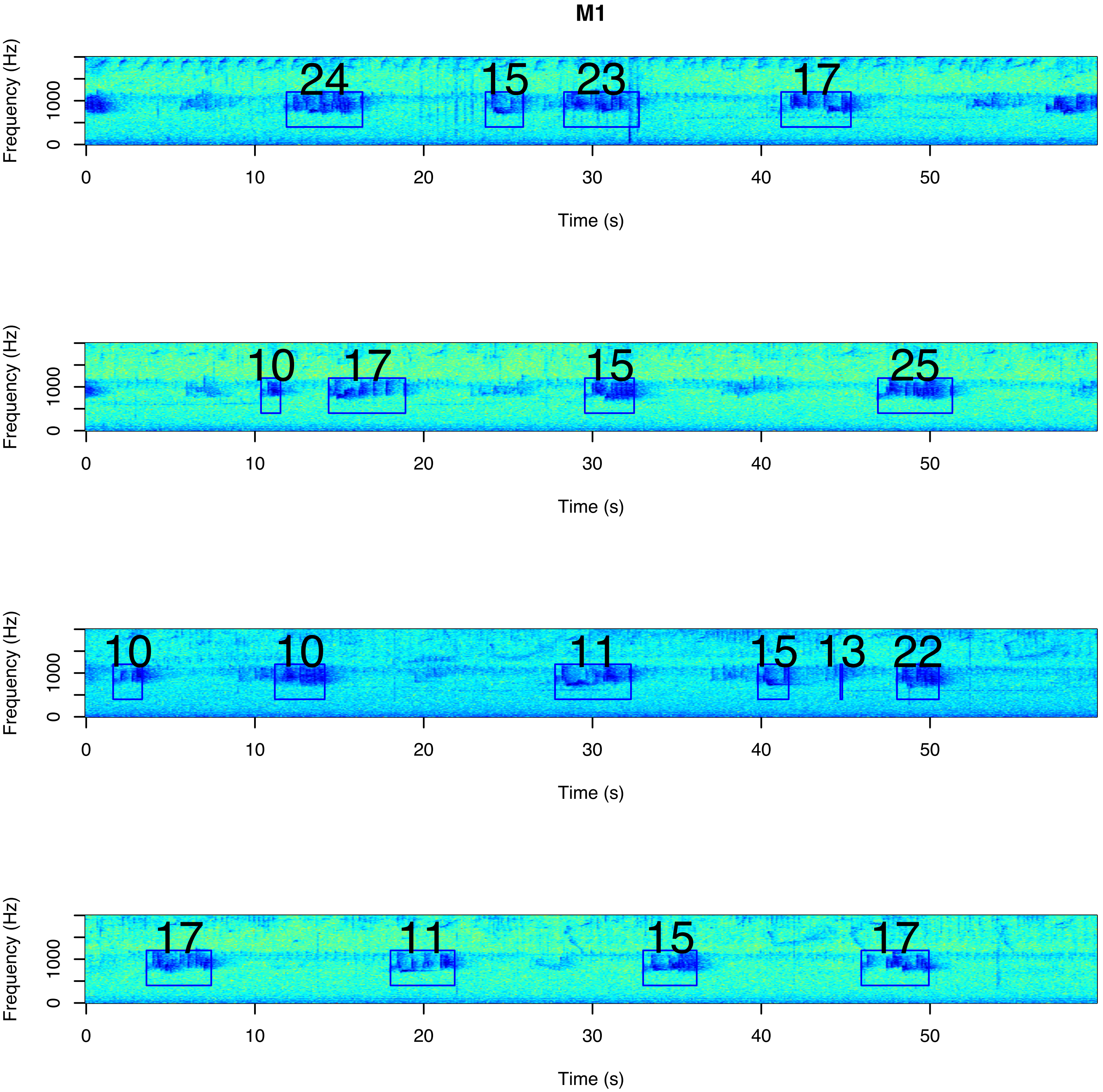
M1
Frequency (Hz)
0
1000
Time (s)
0 10 20 30 40 50
24 15 23 17
10 17 15 25
10 10 11 15 13 22
17 11 15 17

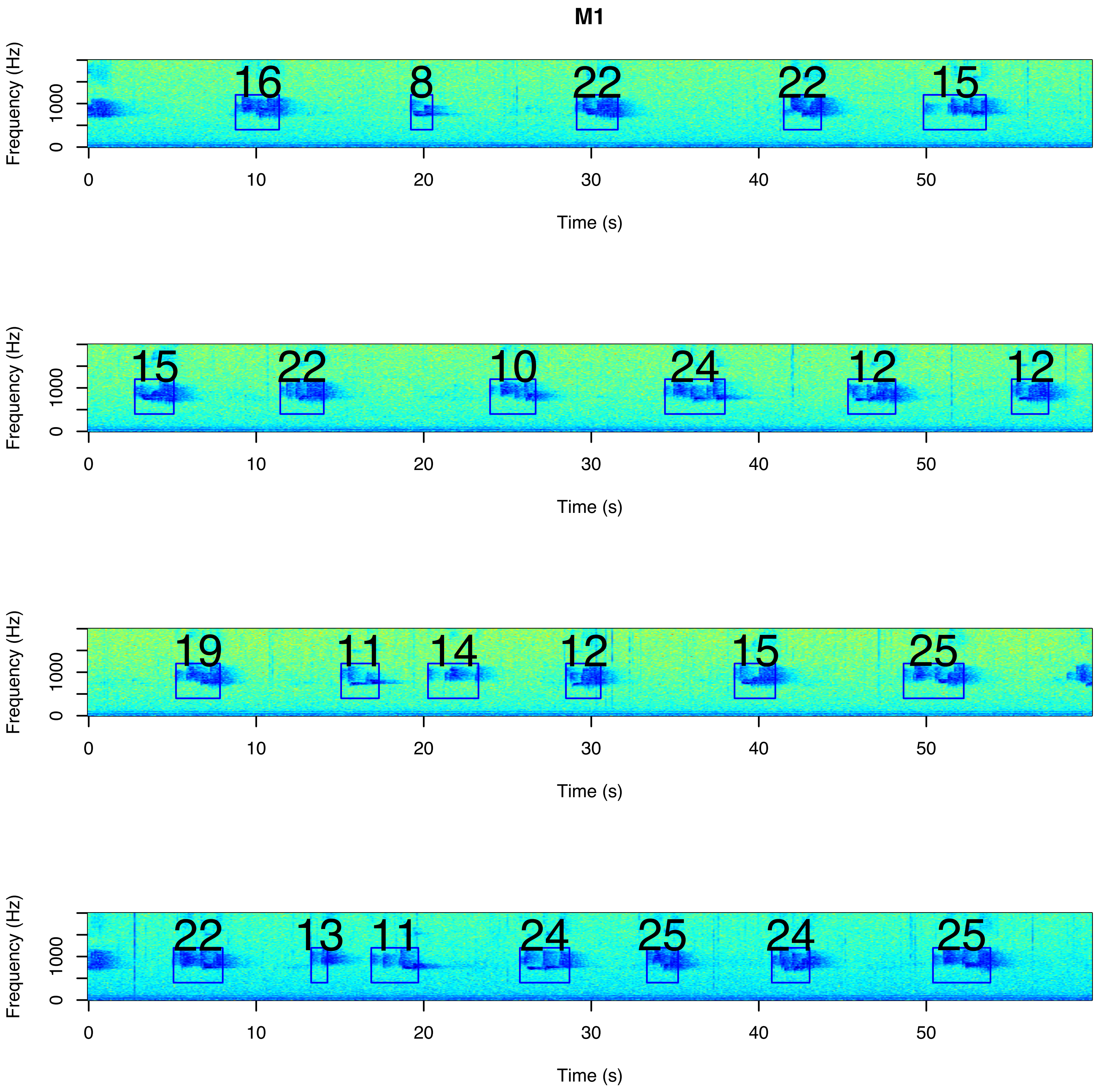
M1
Frequency (Hz)
0
1000
Time (s)
0 10 20 30 40 50
16 8 22 22 15
15 22 10 24 12 12
19 11 14 12 15 25
22 13 11 24 25 24 25

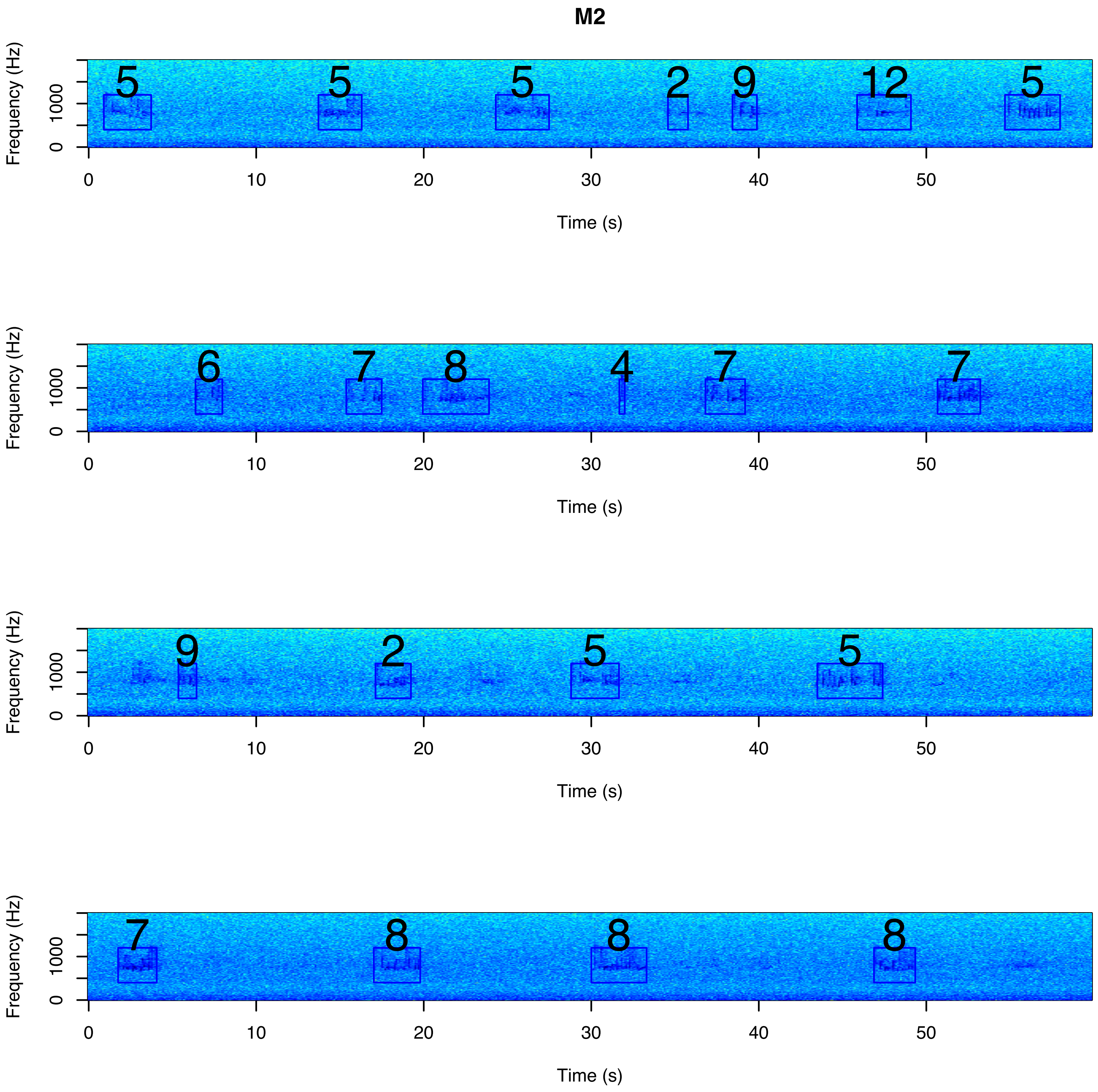

M2
Frequency (Hz)
0
1000
Time (s)
0
10
20
30
40
50
5
5
5
2
9
12
5
6
7
8
4
7
7
9
2
5
5
7
8
8
8

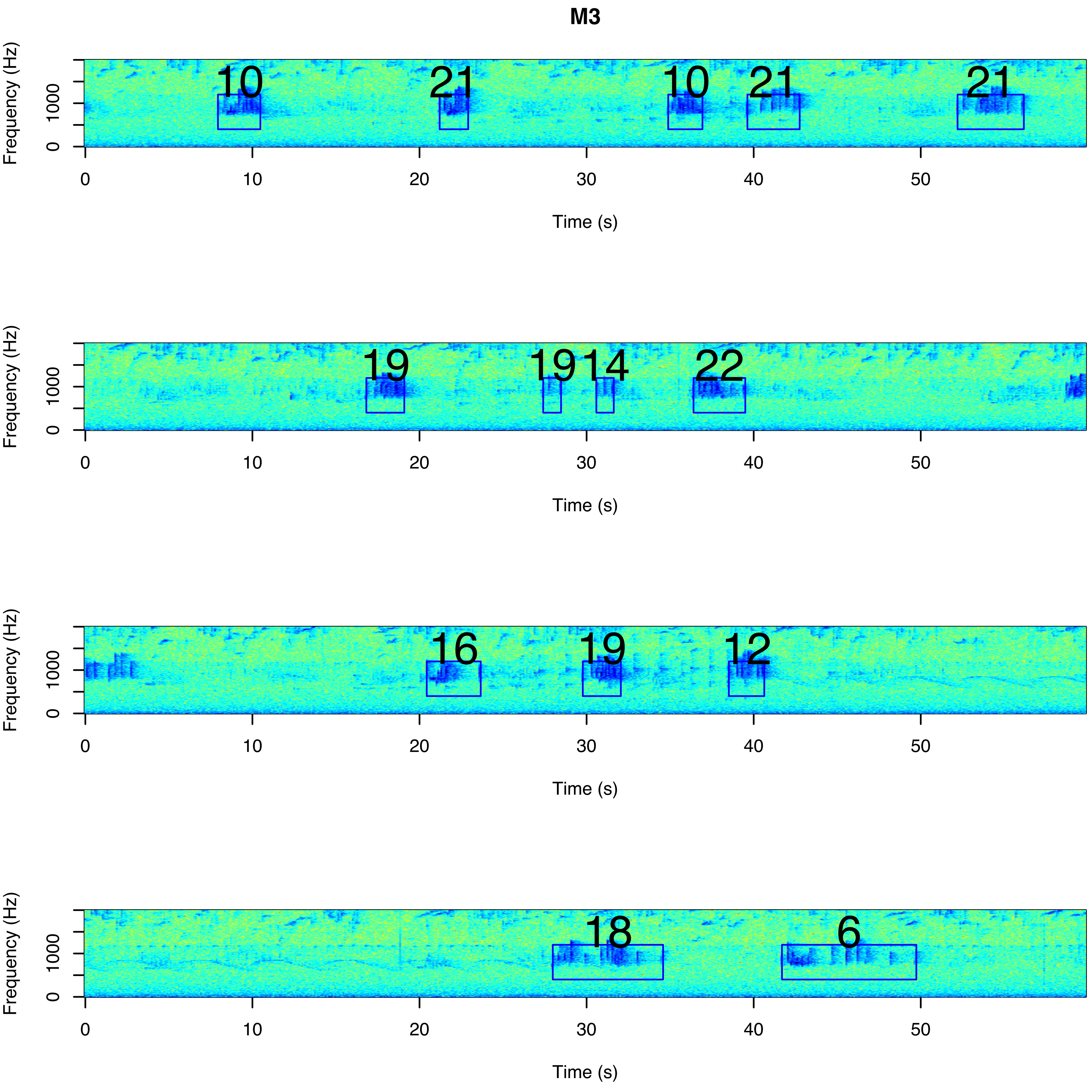
M3
Frequency (Hz)
0
1000
Time (s)
0
10
20
30
40
50
10
21
10
21
21
19
19
14
22
16
19
12
18
6

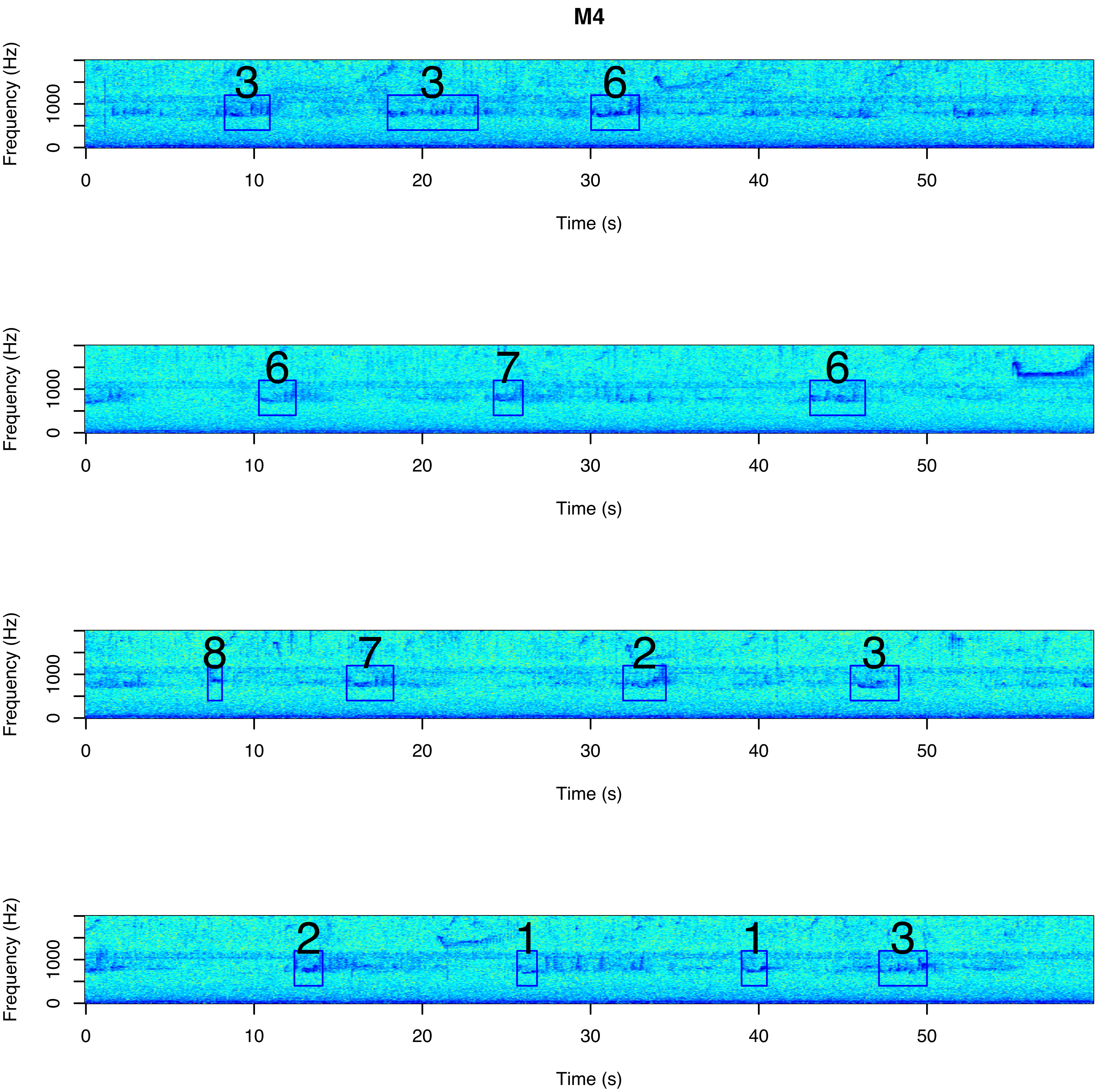
M4
Frequency (Hz)
0
1000
Time (s)
0
10
20
30
40
50
3
3
6
6
7
6
8
7
2
3
2
1
1
3

D1

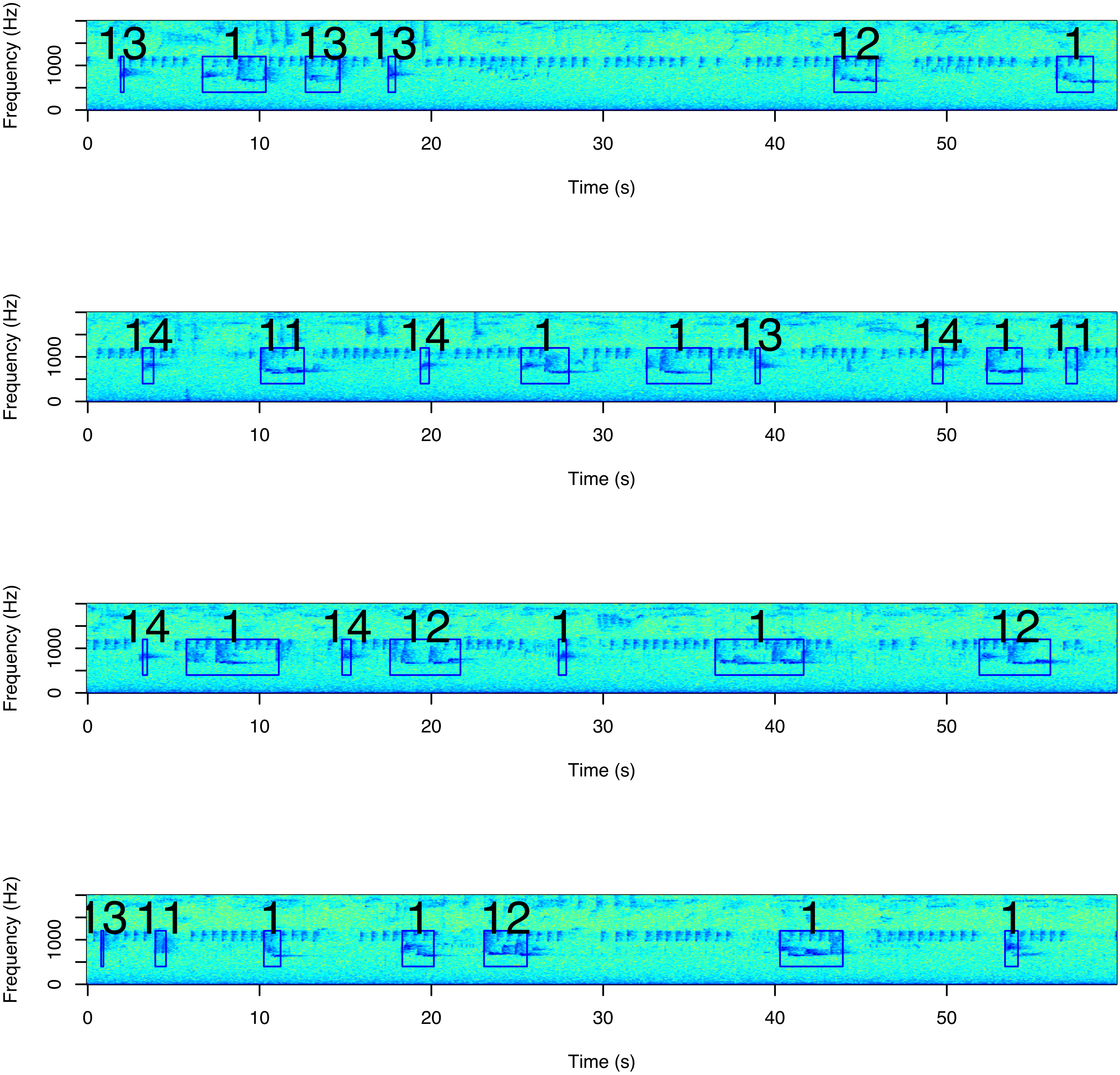
Frequency (Hz)
0
1000
Time (s)
0
10
20
30
40
50
13 1 13 13 12 1
14 11 14 1 1 13 14 1 11
14 1 14 12 1 1 12
13 11 1 1 12 1 1

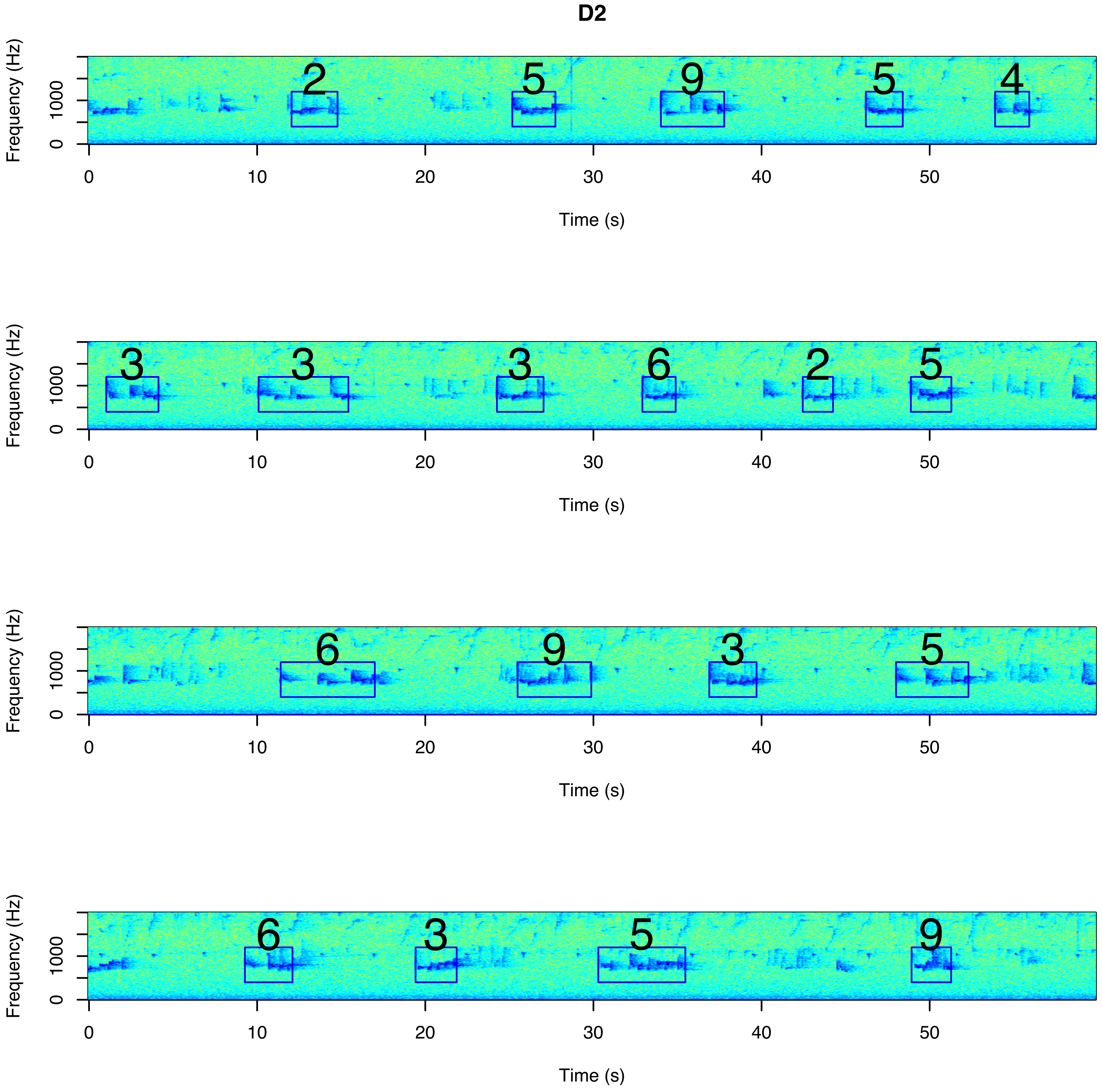
D2
Frequency (Hz)
0
1000
Time (s)
0
10
20
30
40
50
2
5
9
5
4
3
3
3
6
2
5
6
9
3
5
6
3
5
9

D2

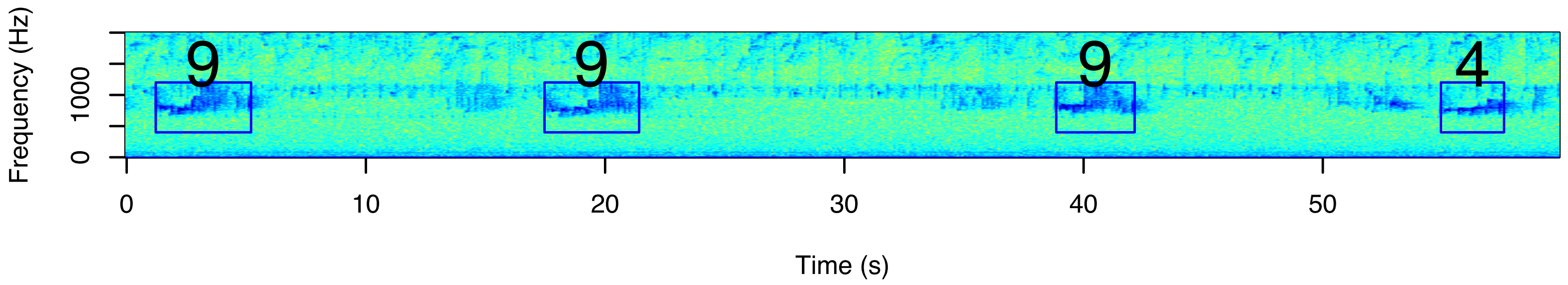

9
9
9
4
Frequency (Hz)
0
1000
0
10
20
30
40
50
Time (s)

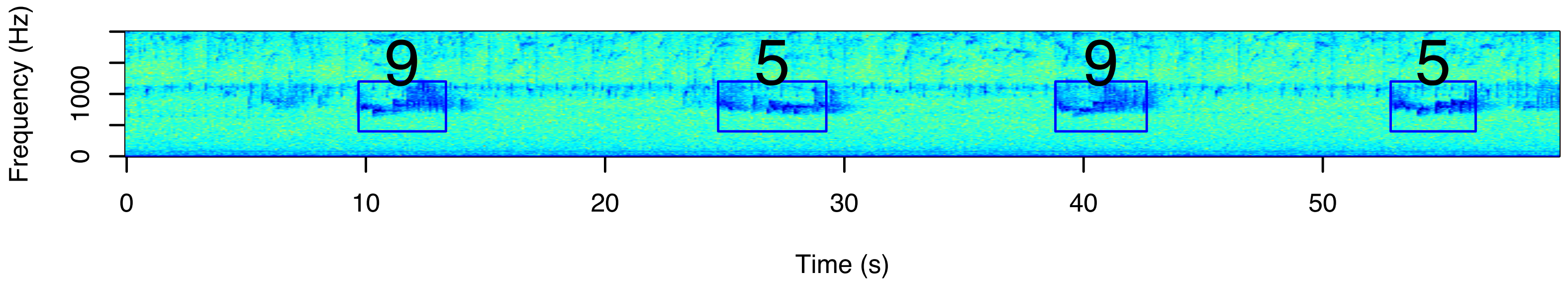

9
5
9
5
Frequency (Hz)
0
1000
0
10
20
30
40
50
Time (s)

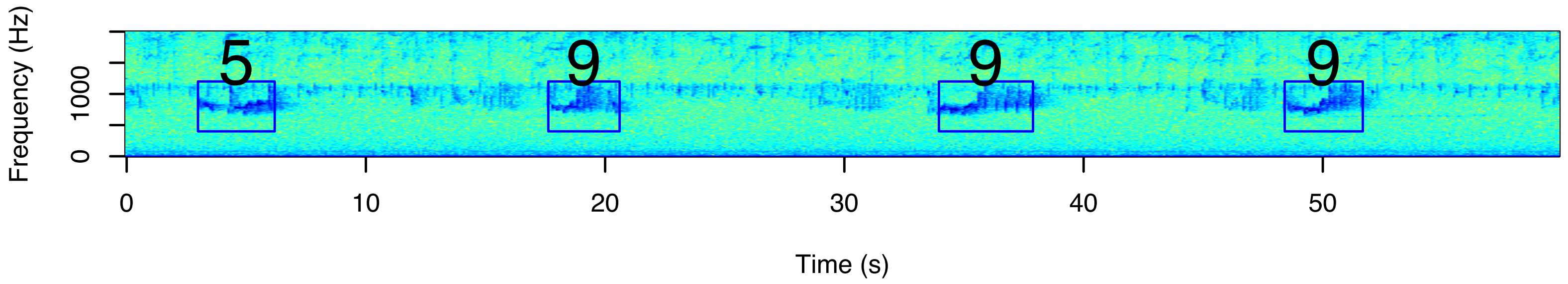

5
9
9
9
Frequency (Hz)
0
1000
0
10
20
30
40
50
Time (s)

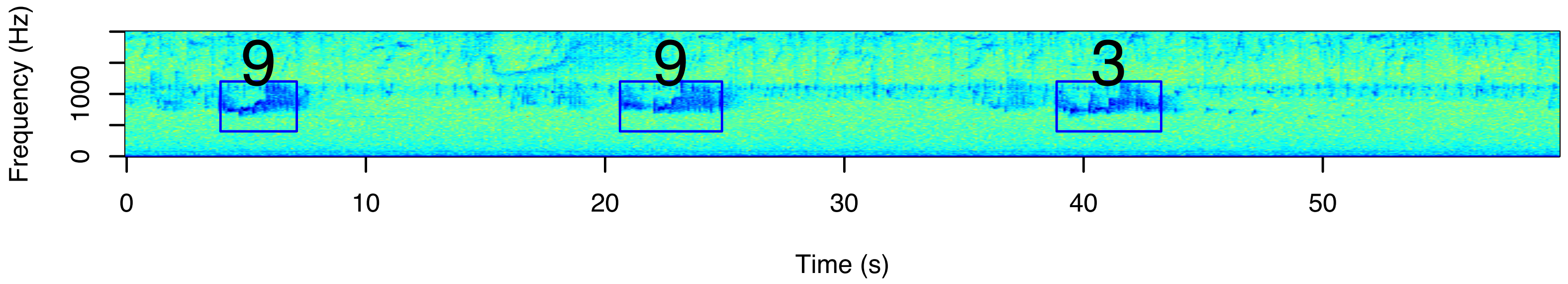

9
9
3
Frequency (Hz)
0
1000
0
10
20
30
40
50
Time (s)

D3

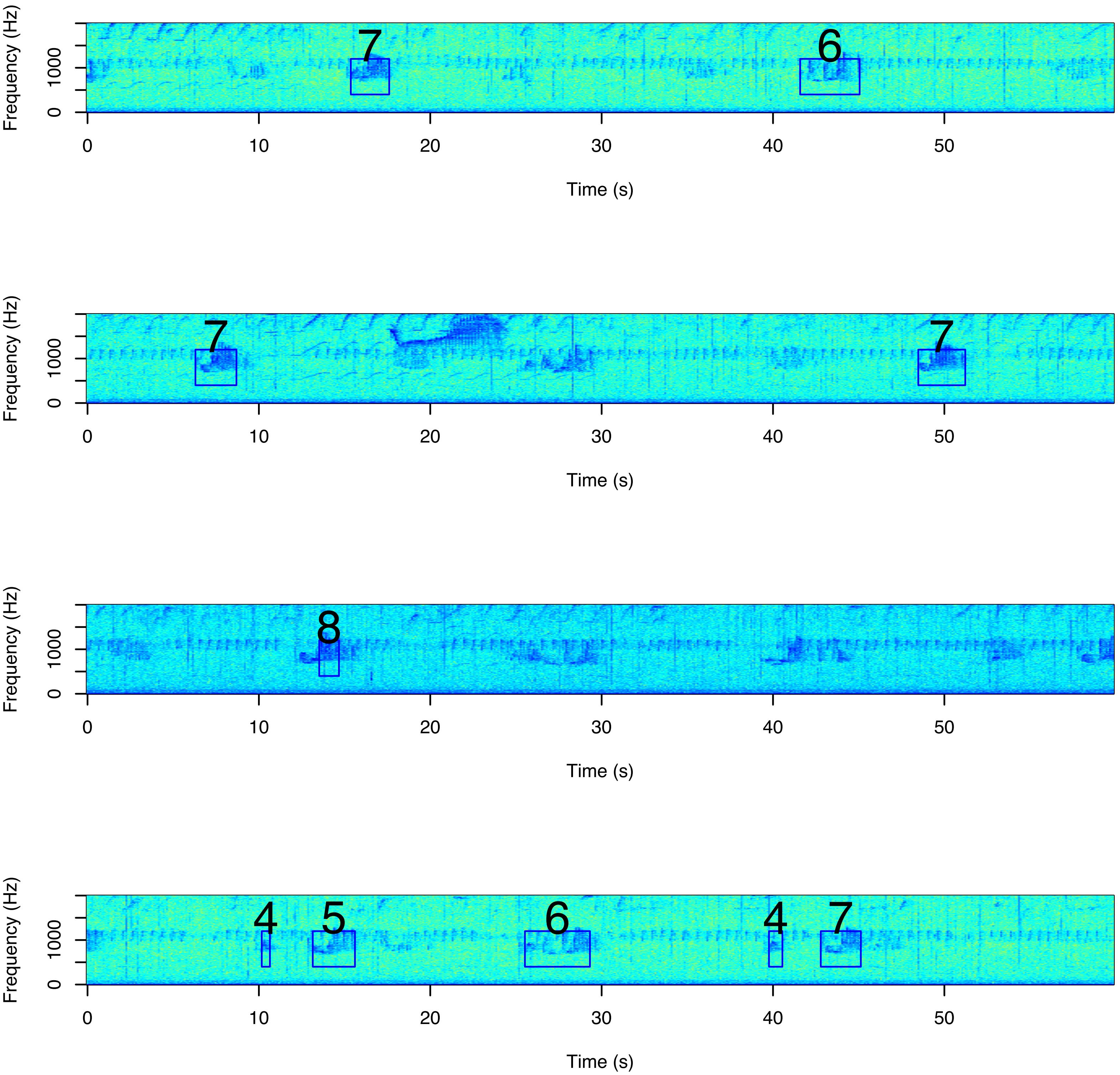
Frequency (Hz)
0
1000
7
6
0
10
20
30
40
50
Time (s)
Frequency (Hz)
0
1000
7
7
0
10
20
30
40
50
Time (s)
Frequency (Hz)
0
1000
8
0
10
20
30
40
50
Time (s)
Frequency (Hz)
0
1000
4
5
6
4
7
0
10
20
30
40
50
Time (s)

D4

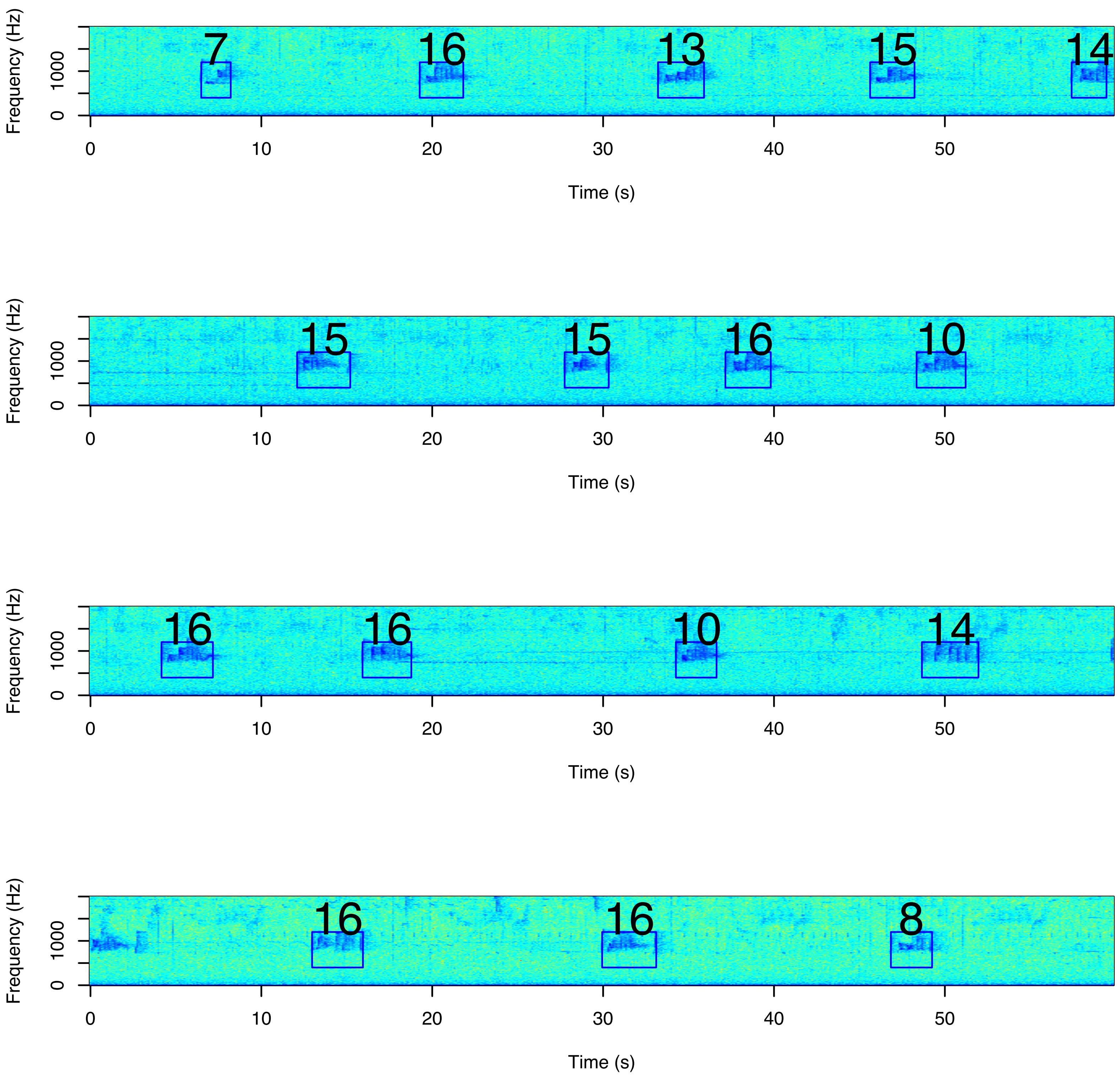

Frequency (Hz)
0
1000
7
16
13
15
14
0
10
20
30
40
50
Time (s)
Frequency (Hz)
0
1000
15
15
16
10
0
10
20
30
40
50
Time (s)
Frequency (Hz)
0
1000
16
16
10
14
0
10
20
30
40
50
Time (s)
Frequency (Hz)
0
1000
16
16
8
0
10
20
30
40
50
Time (s)

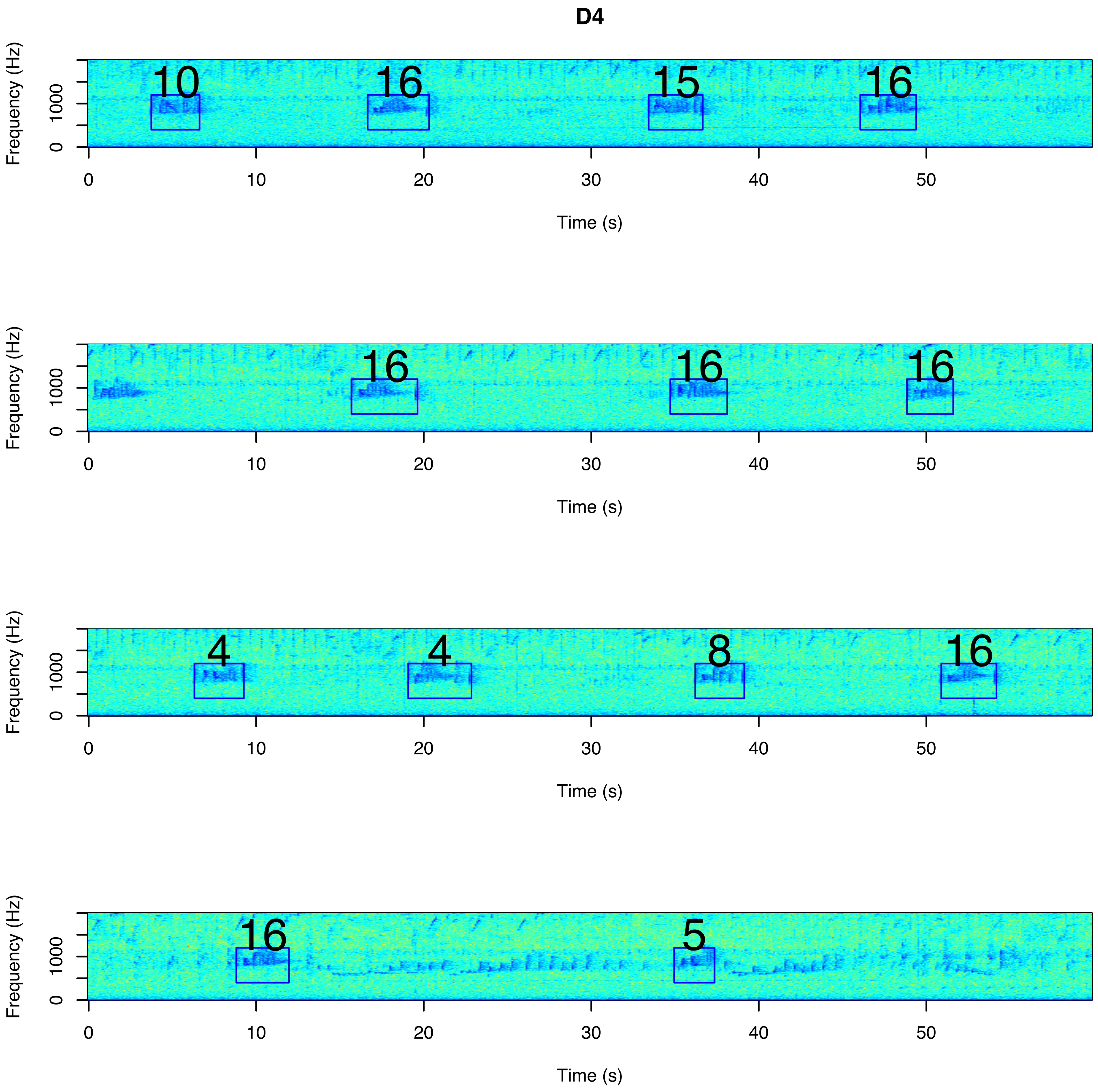
D4
Frequency (Hz)
0
1000
Time (s)
0
10
20
30
40
50
10
16
15
16
16
16
16
4
4
8
16
16
5

D5

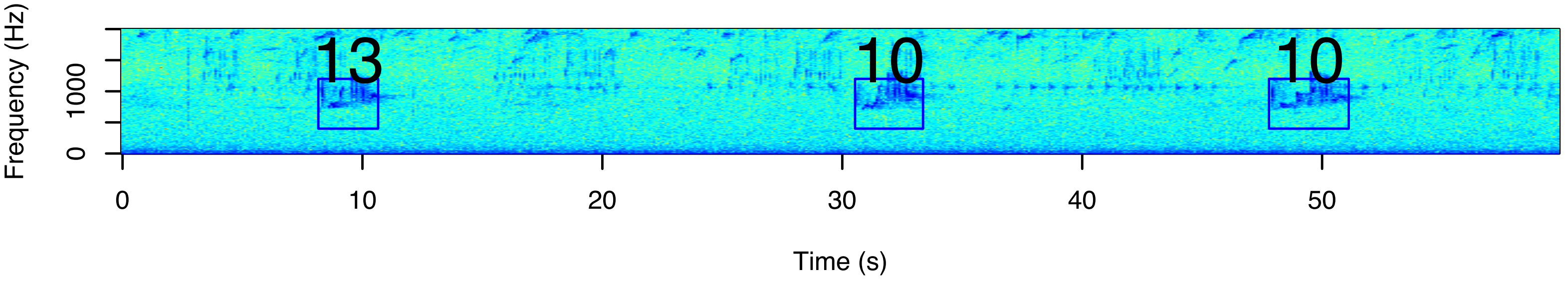

Frequency (Hz)
0
1000
13
10
10
0
10
20
30
40
50
Time (s)

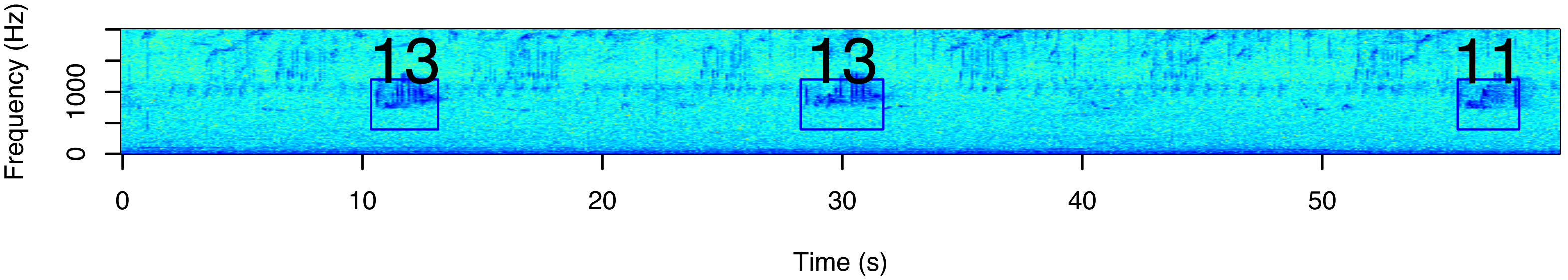

Frequency (Hz)
0
1000
13
13
11
0
10
20
30
40
50
Time (s)

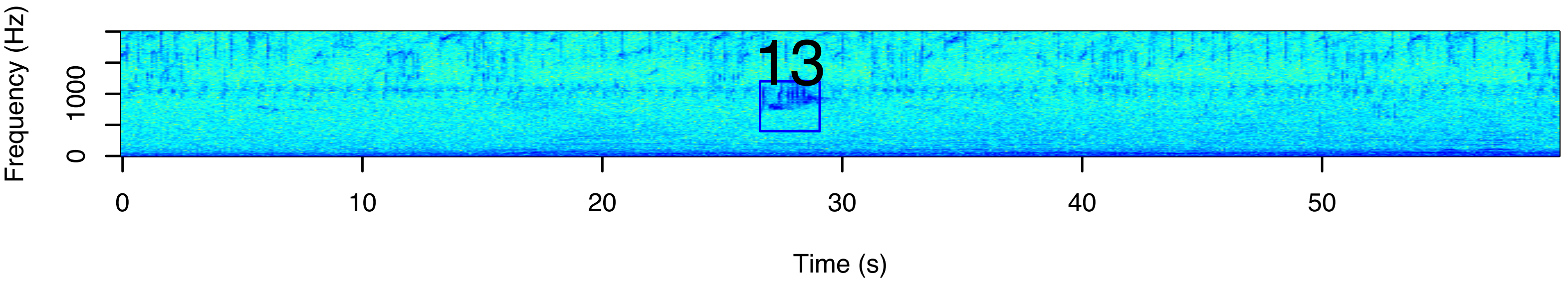

Frequency (Hz)
0
1000
13
0
10
20
30
40
50
Time (s)

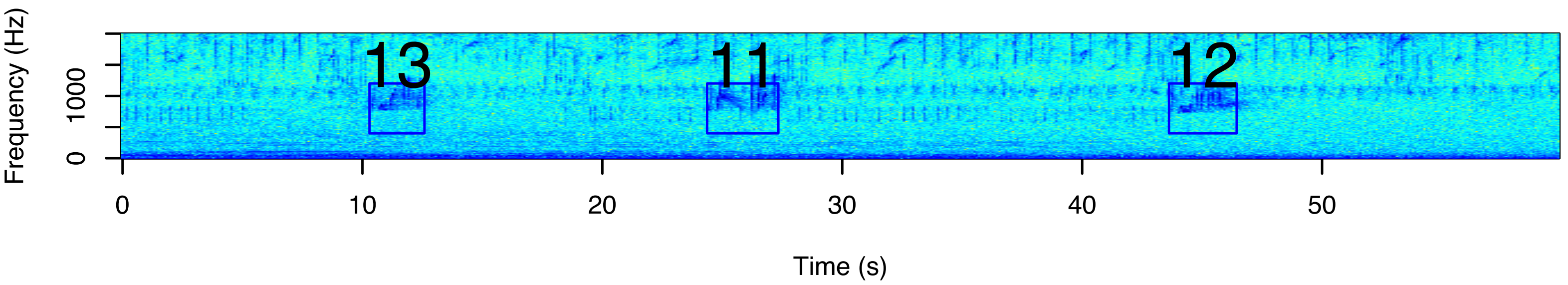

Frequency (Hz)
0
1000
13
11
12
0
10
20
30
40
50
Time (s)

D6

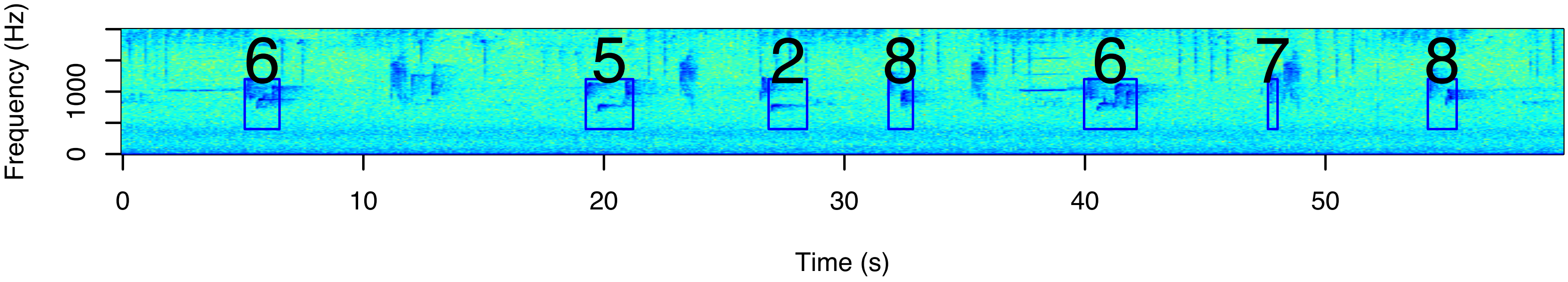

Frequency (Hz)
0
1000
6
5
2
8
6
7
8
0
10
20
30
40
50
Time (s)

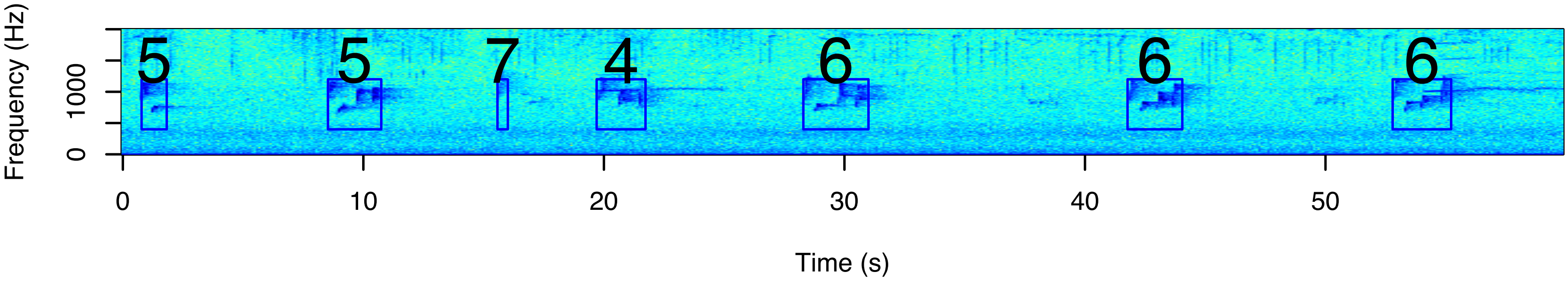

Frequency (Hz)
0
1000
5
5
7
4
6
6
6
0
10
20
30
40
50
Time (s)

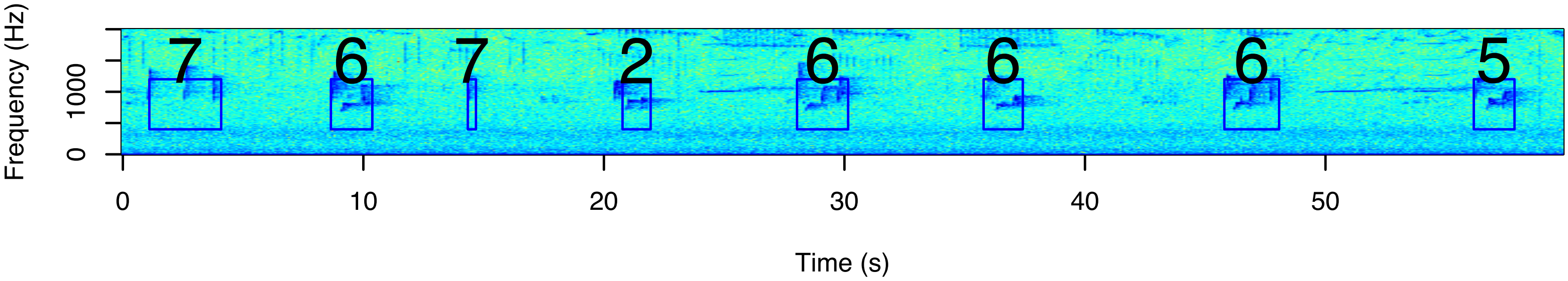

Frequency (Hz)
0
1000
7
6
7
2
6
6
6
5
0
10
20
30
40
50
Time (s)

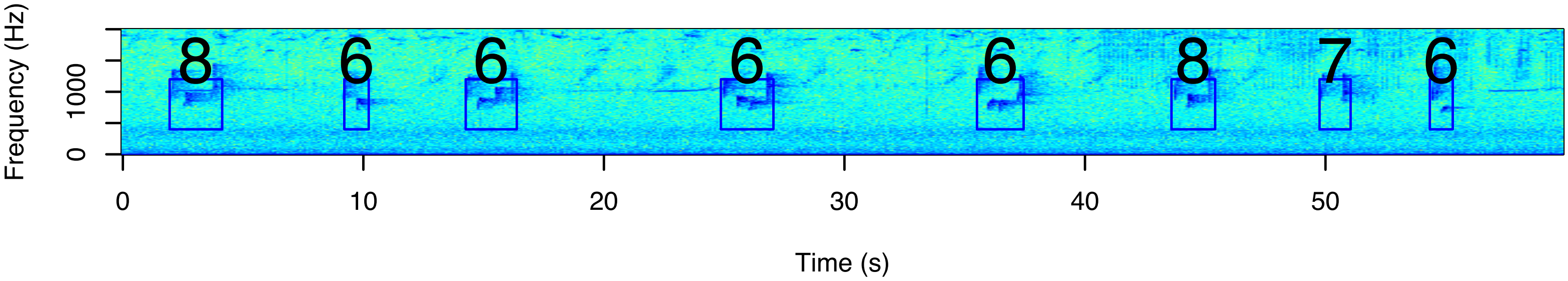

Frequency (Hz)
0
1000
8
6
6
6
6
8
7
6
0
10
20
30
40
50
Time (s)

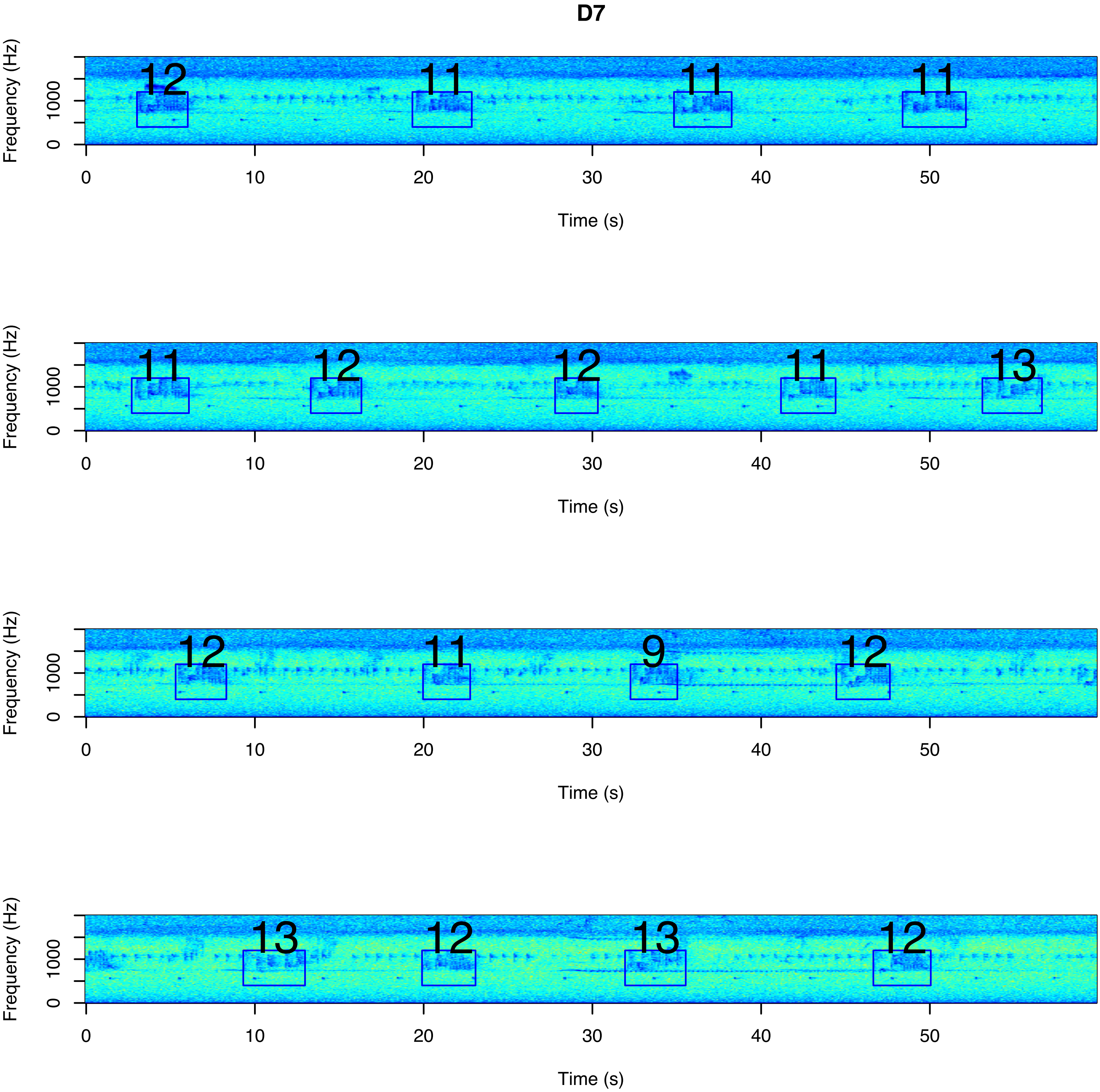
D7
Frequency (Hz)
0
1000
Time (s)
0
10
20
30
40
50
12
11
11
11
11
12
12
11
13
12
11
9
12
13
12
13
12

D8

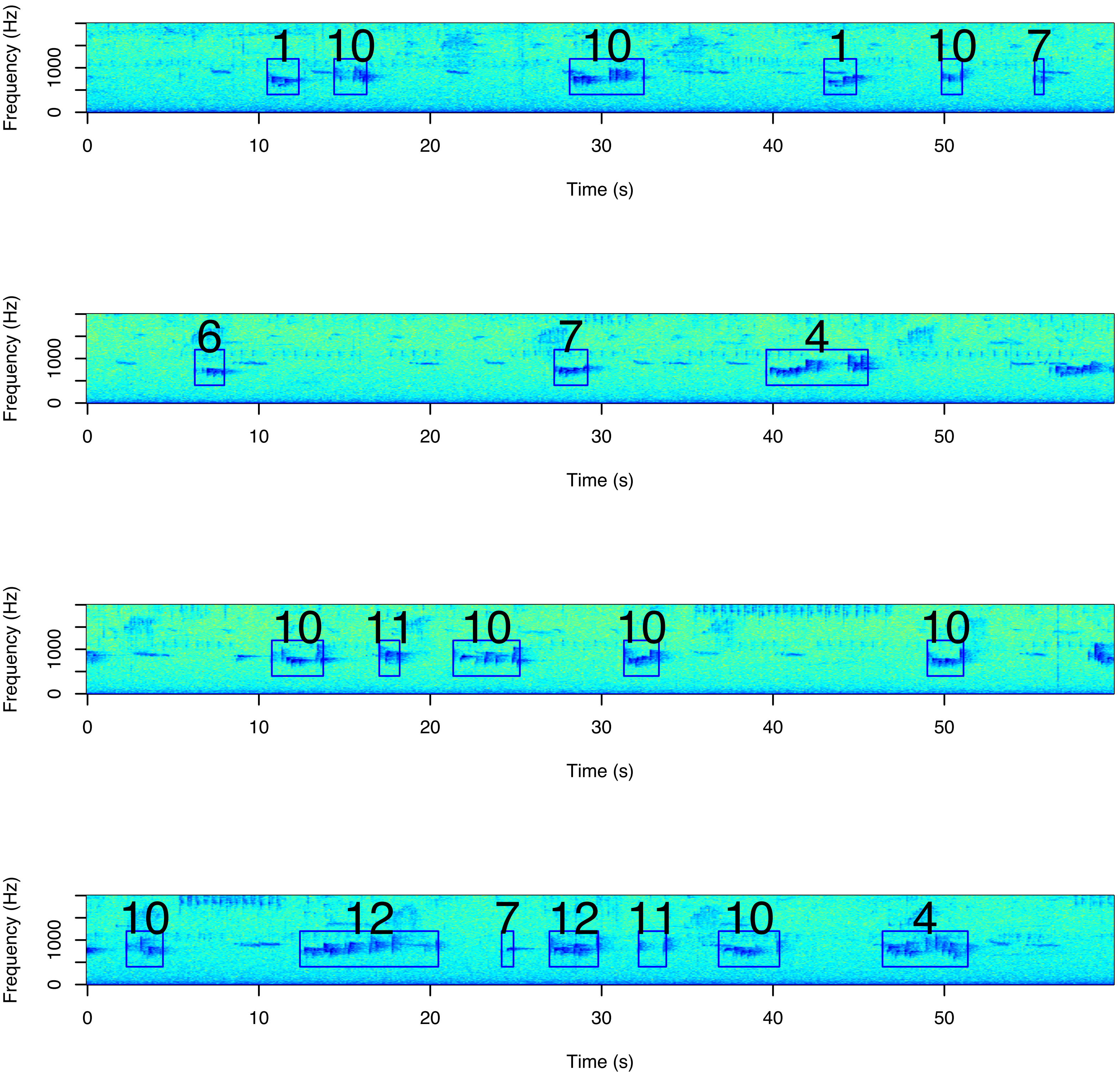

Frequency (Hz)
0
1000
Time (s)
0
10
20
30
40
50
1
10
10
1
10
7
6
7
4
10
11
10
10
10
10
12
7
12
11
10
4

D9

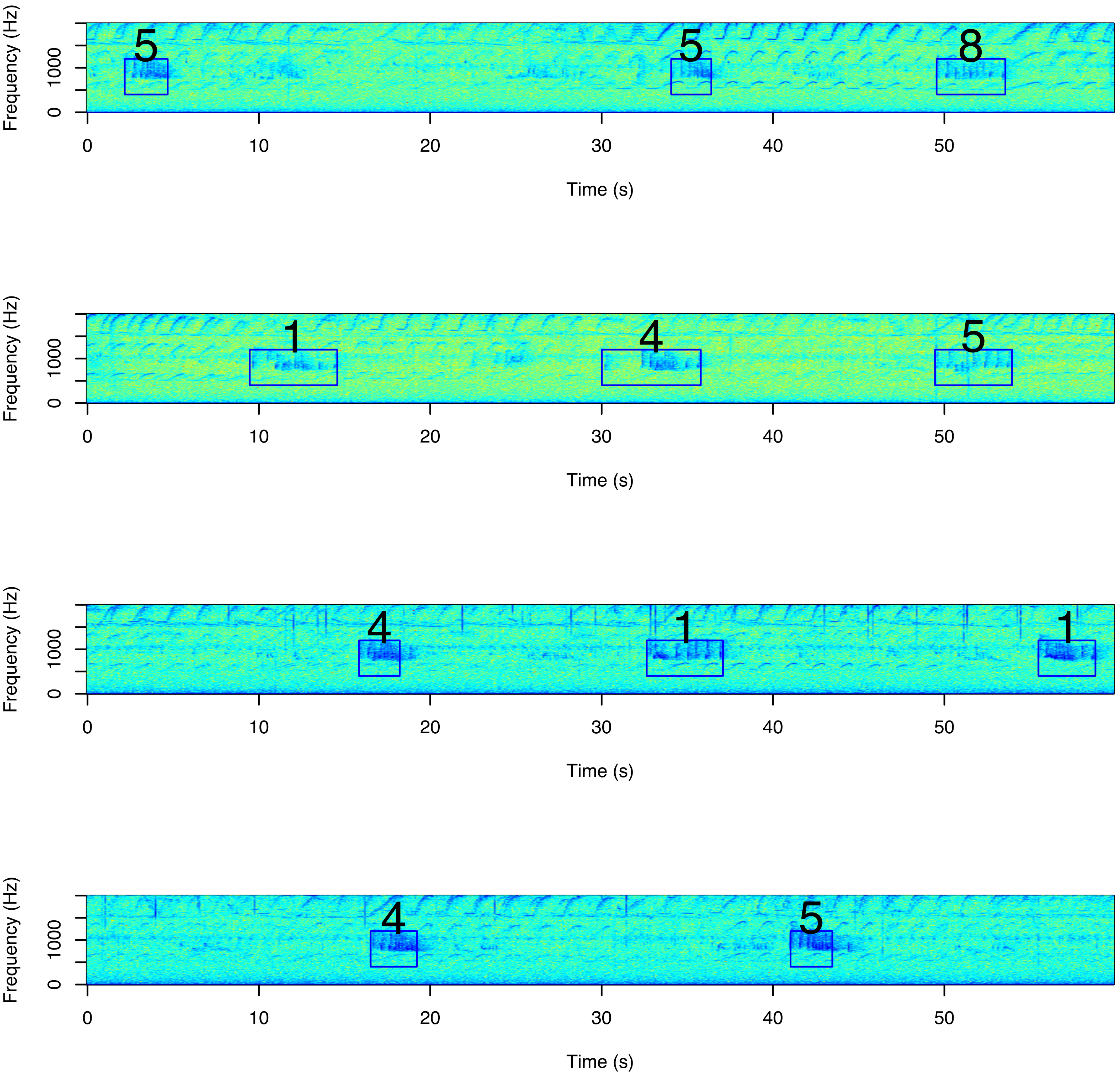

Supplement: Exemplary unsupervised phrase type classification [file rsos200151supp1.pdf]
